# WNT5A as a therapeutic target in breast cancer

**DOI:** 10.1007/s10555-018-9760-y

**Published:** 2018-08-31

**Authors:** Chandra Prakash Prasad, Mansi Manchanda, Purusottam Mohapatra, Tommy Andersson

**Affiliations:** 1 Cell and Experimental Pathology, Department of Translational Medicine, Lund University, CRC, Malmö, Sweden; 2 Department of Medical Oncology (Laboratory), Dr. B.R. Ambedkar IRCH, All India Institute of Medical Sciences, Ansari Nagar, New Delhi, India

**Keywords:** WNT5A, Breast cancer, Cell migration and invasion, Metastasis, Foxy5

## Abstract

Despite the clinical development of novel adjuvant and neoadjuvant chemotherapeutic drugs, metastatic breast cancer is one of the leading causes of cancer-related death among women. The present review focuses on the relevance, mechanisms, and therapeutic potential of targeting WNT5A as a future anti-metastatic treatment strategy for breast cancer patients by restoring WNT5A signaling as an innovative therapeutic option. WNT5A is an auto- and paracrine β-catenin-independent ligand that has been shown to induce tumor suppression as well as oncogenic signaling, depending upon cancer type. In breast cancer patients, WNT5A protein expression has been observed to be significantly reduced in between 45 and 75% of the cases and associated with early relapse and reduced disease-free survival. WNT5A triggers various downstream signaling pathways in breast cancer that primarily affect tumor cell migration and invasion. The accumulated *in vitro* results reveal that treatment of WNT5A-negative breast cancer cells with recombinant WNT5A caused different tumor-suppressive responses and in particular it impaired migration and invasion. The anti-migratory/invasive and anti-metastatic effects of reconstituting WNT5A signaling by the small WNT5A mimicking peptide Foxy5 form the basis for two successful clinical phase 1-studies aiming at determining safety and pharmacokinetics as well as defining dose-level for a subsequent phase 2-study. We conclude that re-installation of WNT5A signaling is an attractive and promising anti-metastatic therapeutic approach for future treatment of WNT5A-negative breast cancer patients.

## Introduction

Members of the wingless-type MMTV integration site (*WNT*) multigene family encode secreted extracellular signaling proteins that regulate several developmental processes, including cell polarity, motility, and patterning, during embryogenesis and tissue homeostasis [1]. In humans, the WNT family consists of 19 highly conserved lipid-modified secreted glycoproteins that exert their effect by interacting with different receptors/co-receptors including frizzled receptors (FZD), low-density lipoprotein receptor-related proteins (LRP5/6), receptor tyrosine kinase-like orphan receptor family (ROR), and the related receptor tyrosine kinase (RYK) [2]. The majority of WNT proteins vary in size between 38 and 43 kDa, they contain up to 22 conserved cysteine residues, and are significantly glycosylated and lipid modified [3]. WNT proteins are hydrophobic in nature and mostly tethered to cell membranes and the extracellular matrix [4]. Furthermore, apart from being hydrophobic, the WNT5A molecule has a heparin sulfate-binding domain that promotes its binding to heparin sulfate proteoglycans (HSPGs). These properties of secreted glycosylated lipid-modified WNT proteins limit their ability to diffuse freely through the extracellular aqueous microenvironment [5, 6]. Instead, WNT-expressing cells secrete WNT proteins in the extracellular environment to form concentration gradients to which the secreting cell or its neighboring cells, expressing the cognate receptors, respond in a concentration-dependent auto- or paracrine manner.

WNT proteins have been classified on the basis of their ability to morphologically change and alter the growth pattern of C57MG mammary epithelial cells [7]. Highly transforming WNT1, WNT2, WNT3, and WNT3A elicit strong morphological alterations; moderately transforming WNT6 and WNT7A induces weak morphological changes; and WNT4, WNT5A, WNT5B, and WNT7B are unable to induce any morphological changes in C57MG cells, highlighting the divergent roles of different WNT proteins, despite their structural similarity [8]. WNT proteins have also been grouped as classical WNTs (WNT1, WNT3A, WNT8, and WNT8B) that activate the β-catenin-dependent (canonical) pathway and non-classical WNTs (WNT4, WNT5A, WNT11) that induce β-catenin-independent (non-canonical) pathways [9]. The β-catenin-dependent pathway is initiated by the binding of WNT to the membrane-bound FZD receptors and co-receptor LRP5 or 6 that antagonizes the activity of the destruction complex consequently stabilizing the β-catenin that then bind to specific transcription factors (i.e*.*, T cell factor/lymphoid enhancer binding factor TCF/LEF) and induce transcription of WNT-responsive genes [10]. Some members of the WNT family, such as WNT5A, signal *via* alternative, β-catenin-independent mechanisms, including calcium/calmodulin-dependent kinase (WNT/Ca^2+^) [11] and the planar cell polarity (PCP) pathways that antagonize β-catenin-dependent signaling [12]. The focus of this review will be on the β-catenin-independent ligand WNT5A. *WNT5A* is one of the extensively studied members of the WNT gene family. WNT5A functions as a morphogen in a variety of tissues and regulates various cellular processes, such as cell proliferation and differentiation, during early embryonic developmental stages. WNT5A^−/−^ mice exhibit several developmental defects, such as dwarfism; shortened limbs and tails; lungs with foreshortened trachea; facial, ear, and genital abnormalities; and ventricular septal defects. These mice die shortly after birth [13, 14]. WNT5A is also involved in mammary gland development and inhibition of ductal extension and branching in the mammary gland [15].

The human *WNT5A* gene was mapped to chromosome 3p14-p21. The gene consists of five exons, with the last exon spanning a 3′-untranslated region of approximately 6.5 kilobase pairs containing multiple polyadenylation sites [16]. The *WNT5A* gene has been shown to encode two different transcripts, a long WNT5A-L isoform, and a short WNT5A-S isoform. These two distinct mRNAs have different translational start sites due to different upstream promoter sequences. In breast cancer, WNT5A-S mRNA expression was elevated, while WNT5A-L mRNA was decreased, thereby suggesting that if these different mRNA isoforms are translated to different WNT5A isoforms that they might have distinct biochemical functions. However, so far, such a possibility has not been demonstrated at the protein level nor has it been addressed *in vivo* [2]. Additionally, the WNT5A transcript contains several AU-rich sequences in its 3′-untranslated region, highlighting the importance of several RNA binding proteins as its potential regulators. In breast cancer, WNT5A has been shown to be regulated at the post-transcriptional level *via* HuR, a member of embryonic lethal abnormal vision (ELAV)-like protein family that binds to evolutionary conserved AU-rich motifs in the untranslated region of its mRNA and suppresses translation [17]. The mature human WNT5A protein shares > 99% homology to mouse and > 93% to other WNT5A proteins reported in other species [16].

Previous studies have demonstrated that ectopic treatment with commercially available full-length recombinant WNT5A (rWNT5A) can elucidate both an oncogenic and a tumor-suppressive role of WNT5A depending on the cancer type. These findings suggest that the interaction between WNT5A and its receptors determine the type of downstream outcome in different types of cancer. Loss of WNT5A has been associated with poor prognosis in a number of different cancer types such as breast cancer, colon cancer, neuroblastoma, and leukemia, thus suggesting a tumor-suppressive function of WNT5A in these tumor subtypes [18]. In the present review, we will discuss how WNT5A has emerged as a tumor suppressor in breast cancer, and how re-activating its signaling pathway in breast cancer cells can be an attractive alternative to curb this dreaded disease.

## WNT5A expression in breast cancer

WNT5A expression has been studied in human breast cancer tissue by various investigators at both the transcriptional and protein level. However, it should be noted that WNT5A mRNA levels have been documented not to correlate to the WNT5A protein levels in breast cancer tissue, since the translation of WNT5A mRNA can be regulated *via* its untranslated 3′-region, thereby generating discrepancies between WNT5A mRNA levels and protein expression [3, 17, 19, 20]. A similar discrepancy between WNT5A mRNA and protein levels has also been reported in hepatocellular carcinoma [21]. Hence, it can be misleading to draw conclusions based on correlations between WNT5A mRNA and clinicopathological parameters or clinical response. Hence, we have opted not to discuss those mRNA-based studies in the current review to minimize confusion.

The first immunohistochemical study on WNT5A was performed on 96 patients with primary invasive ductal carcinoma (IDCs) using a specific anti-WNT5A antibody [22]. Approximately, 45% of these IDCs showed loss or low expression of the WNT5A protein that was associated with an absence of estrogen, as well as progesterone receptors and increasing histological grade. Additionally, 83 patients (with longer follow-up) were further incorporated in the same study to investigate the prognostic significance of WNT5A protein expression [22]. The authors reported loss of or reduced WNT5A protein expression in 78% of patients with recurrence but only in 35% of those without recurrence. Furthermore, it was demonstrated that recurrence-free survival was significantly shorter in patients with no or reduced WNT5A protein expression [22]. Another independent investigation revealed loss of or reduced WNT5A protein expression in the tumor tissue from 56% of the included breast cancer patients [19]. These authors also reported that the presence of WNT5A protein was associated with longer disease survival, while in univariate analyzes, the WNT5A negative group indicated an increased risk of mortality. Additionally, the WNT5A levels in the tumor samples did not correlate with proliferation markers or phosphorylation of the retinoblastoma protein (RB), indicating that the WNT5A protein is not involved in the proliferation of breast cancer [19]. Using specific probes for detecting WNT5A mRNA (no cross-reactivity with WNT5B), the authors also showed that tumors (*n* = 20) expressing significant levels of WNT5A mRNA had no expression of the WNT5A protein, indicating for the first time that WNT5A protein expression could be regulated at the translational level [19]. One such translational mechanism was revealed by Leandersson and co-workers, who demonstrated that the ELAV-like protein HuR can inhibit WNT5A mRNA translation by binding to its conserved AU-rich sequences in 3΄-UTR regions present in the WNT5A transcript [17].

Later, the loss of WNT5A expression in 146 tumors (40%) in a pre-menopausal cohort of 361 samples with a median follow-up time of 14 years was reported. WNT5A emerged as an independent prognostic marker in estrogen receptor-positive tumors and was associated with good prognosis in Luminal A tumors. Furthermore, reduced WNT5A expression significantly correlated with young age, estrogen receptor negativity, and the triple-negative breast cancer (TNBC) phenotype [23]. Several other researchers also demonstrated a similar loss of WNT5A protein in TNBC patients by immunohistochemical staining. Borcherding and co-workers showed loss of WNT5A protein in 75% of TNBC patients [24]. Similarly, other investigators have recently demonstrated that decreased expression of WNT5A is a prognostic factor in TNBC patients by showing that patients with reduced expression of WNT5A in their tumor tissue have poor recurrence-free survival [25]. Additionally, it has been shown that early breast tumors and pre-neoplastic lesions express WNT5A protein that decreases in late-stage tumors and lung metastases [24]. Overall, studies of WNT5A protein expression in breast cancer tissue reveal that it functions as a tumor suppressor in this type of cancer, where its loss correlates with breast cancer progression and metastasis. As a ligand, WNT5A triggers various signaling cascades by binding to its cognate receptors or receptor/co-receptor complexes. Thus, in order to understand the mechanism whereby reduced expression of the WNT5A protein leads to breast cancer progression, one has to understand how WNT5A signaling affects breast cancer cell behavior.

## WNT5A signaling regulates breast cancer cell migration and invasion

The metastatic spread of breast cancer cells is a sequential series of events involving reduced cell-cell and cell-basement membrane interactions, increased tumor cell migration, and invasion through the basement membrane and entrance into the circulation *via* lymph and/or blood vessels to finally reach distant organ sites where the tumor cells can invade and proliferate and give rise to a metastatic foci. WNT5A has been shown to control various signaling pathways involved in the regulation of breast cancer cell migration and invasion and thus, the metastatic process. In the present section, we will discuss WNT5A signaling and how it can affect the functional responses of breast cancer cells with a focus on their migration and invasion.

Initial findings on WNT5A suggested that its mRNA expression in human mammary epithelial HB2 cells could be regulated by cell shape, confluence, and hepatocyte growth factor (HGF). At confluence, WNT5A was upregulated by 30-fold; however, its expression decreased by 3-fold when cells were transferred from a two-dimensional surface (flat cell morphology) to three-dimensional gels (spherical shape morphology) [26]. These findings suggest that WNT5A upregulation upon cell confluence might inhibit cell migration. However, it should be pointed out that these findings were not explored and confirmed in relation to WNT5A protein expression. Using immortalized mammary epithelial cells (HB2), it was demonstrated that overexpression of WNT5A increased binding of mammary epithelial cells to collagen and obliterated HGF triggered migration. In contrast, downregulation of WNT5A in these cells resulted in cell scattering, deregulated cell-collagen interaction, and increased cell migration. The authors further demonstrated that downregulation of WNT5A impairs both cell-to-collagen binding and collagen-induced discoidin domain receptor-1 (DDR1) phosphorylation, suggesting a direct relationship between WNT5A-dependent DDR1-phosphorylation and breast cancer cell adhesion to the collagen matrix [27]. Subsequently, it was shown that the WNT5A-induced increase in DDR1 phosphorylation/activation in mammary epithelial HB2 cell adhesion is mediated *via* activation of the heterotrimeric G_i/o_ protein, as revealed by pertussis toxin experiments. Based on these findings, mastoparan was used to directly activate heterotrimeric G_i/o_ proteins in WNT5A-negative breast cancer cells, resulting in reconstitution of collagen to induce phosphorylation of DDR1 and an increased adhesiveness of these breast cancer cells, thereby proposing the therapeutic notion to use mastoparan to reinstall WNT5A signaling in WNT5A-negative breast cancer [28]. As the expression of WNT5A protein predicts longer disease-free survival in breast cancer patients, its metastasis-suppressing activity might at least in part be due to increased DDR1-dependent cell adhesion and decreased migration. Another important mechanism that could contribute to the anti-invasive property of WNT5A signaling in breast cancer is the activation status of the transcription factor nuclear factor associated with T cells (NFAT). The activity of this transcription factor has been shown to directly correlate with breast cancer migration and invasion [29, 30]. Interestingly, WNT5A has been shown to induce Yes/Cdc42/CK1α signaling that counteracts NFAT activity [31].

Interestingly, observations made during studies of mammary gland development lend interesting clues to the situation in breast cancer [32]. It was demonstrated that WNT5A is essential for transforming growth factor-β (TGF-β) to impair mammary branching during mammary gland development. Pellets of TGF-β placed in front of the end bud inhibited ductal extension together with decreased end bud size; however, the *WNT5A*^−/−^ epithelium was unaffected by the inhibitory effects of TGF-β [15]. Also in this developmental context, DDR1 phosphorylation was a downstream target of WNT5A signaling. The described link between TGF-β and WNT5A was later pursued in a separate study, where the authors showed that loss of TGF-β or WNT5A led to nuclear localization and activation of β-catenin and its target genes, suggesting that TGF-β and WNT5A antagonize WNT/β-catenin signaling [33]. This finding is interesting, since activation of WNT/β-catenin signaling plays a crucial role in breast cancer progression and metastasis [34, 35]**.**

In normal breast cells, the majority of β-catenin protein is present on the membrane in the cadherin-catenin complex and maintains cell-cell adhesion. During cancer progression, there is a loss of cell-cell adhesion. In human breast epithelial cells, the WNT5A-CKIα signaling axis has been shown to promote β-catenin/E-cadherin complex formation. However, in breast cancer, loss of WNT5A expression and signaling was associated with a reduced level of membranous β-catenin, suggesting that decreased cell-cell adhesion and increased migration contribute to metastatic disease [36]. Apart from its role in cell-cell adhesion, WNT5A signaling also induced the phosphorylation of Thr34 on the dopamine and cAMP-regulated phosphoprotein of 32 KDa (DARPP-32). Interestingly, the WNT5A triggered phosphorylation of DRAPP-32 was shown to impair MCF7 breast cancer cell migration. The investigators delineated a specific WNT5A signaling axis, i.e., FZD3/G_αs_/cAMP/PKA, to be responsible for the DARPP-32 and CREB-dependent anti-migratory effects in breast cancer cells [37]. A distinct role for the small GTPase Cdc42 in WNT5A signaling in breast cancer cells has been demonstrated. Somewhat surprisingly, it was shown that WNT5A signaling impairs breast cancer migration *via* activation of Cdc42. However, the authors demonstrated that WNT5A-triggered Cdc42 activation inhibits ERK1/2-MMP-9 signaling, resulting in impaired breast cancer migration and invasion [38]. CD44 is a transmembrane glycoprotein that is associated with breast cancer metastasis [39, 40]. It has been shown that WNT5A signaling alters CD44 splicing, resulting in reduced levels of specific variant isoforms, e.g., CD44v4 and CD44v6, in WNT5A-expressing cells [9]. Later, in an independent study, it was shown that WNT5A signaling impairs CD44-AKT signaling, leading to impaired breast cancer cell migration and invasion. CD44 has also been connected to the epithelial-mesenchymal transition (EMT) in cancer cells, which is interesting since EMT has been related to increased migration and invasion of cancer cells. However, it has been shown that the ability of WNT5A signaling to impair breast cancer cell migration and invasion is not related to a reversal of the EMT status of these cancer cells, thus defining the action of WNT5A as an EMT-independent mechanism [41].

To this point, we have only discussed the role of tumor cell-derived WNT5A; however, there is a very interesting study revealing a role of paracrine WNT5A signaling leading to a reduced number of tumor-initiating cells (TIC). Using transcript profiling, the authors of this study demonstrated that tumors derived from basal and luminal TIC showed preferential loss of WNT5A. In addition, overexpression of WNT5A triggered a feed-forward loop to activate SMAD2, *via* RYK and the transforming growth factor β-receptor 1 (TGFβR1), resulting in limited expansion of basal TICs, hence providing another possible but not proven explanation for the tumor-suppressive effects of WNT5A in breast cancer [24].

Apart from migration and invasion, there are reports of the anti-proliferative effect of WNT5A. In a recent study, WNT5A has been shown to repress ribosomal DNA transcription, implying that WNT5A signaling can target RNA synthesis. Using breast cancer cells, the authors demonstrated that WNT5A signaling through segment polarity protein dishevelled homolog (DVL1) represses ribosomal DNA gene transcription and generates a chromatic state resulting in less transcription of rDNA by RNA polymerase I [42].

Considering all these reports, it is certain that WNT5A signaling exerts tumor-suppressive effects in breast cancer. Ectopic expression of WNT5A protein suppresses breast cancer progression by increasing adhesion with concomitant decrease in cell migration and invasion. Whether and to what extent WNT5A signaling also affects breast cancer cell proliferation, as suggested above [42], needs further investigation. However, breast cancer is a heterogeneous disease that may lead to mixed responses to WNT5A signaling in different breast cancer studies. We believe that the effect on β-catenin signaling is a major deciding factor that governs the WNT5A response in breast cancer cells. Therefore, before using any breast cancer cell line model or clinical cohort, investigators should check for the endogenously present WNT/β-catenin signaling pathway. For example, the WNT/β-catenin signaling pathway is hyperactive in MDA-MB-231 breast cancer cells, whereas it is only moderately active in MCF7 breast cancer cells [43, 44].

**Fig. 1:**
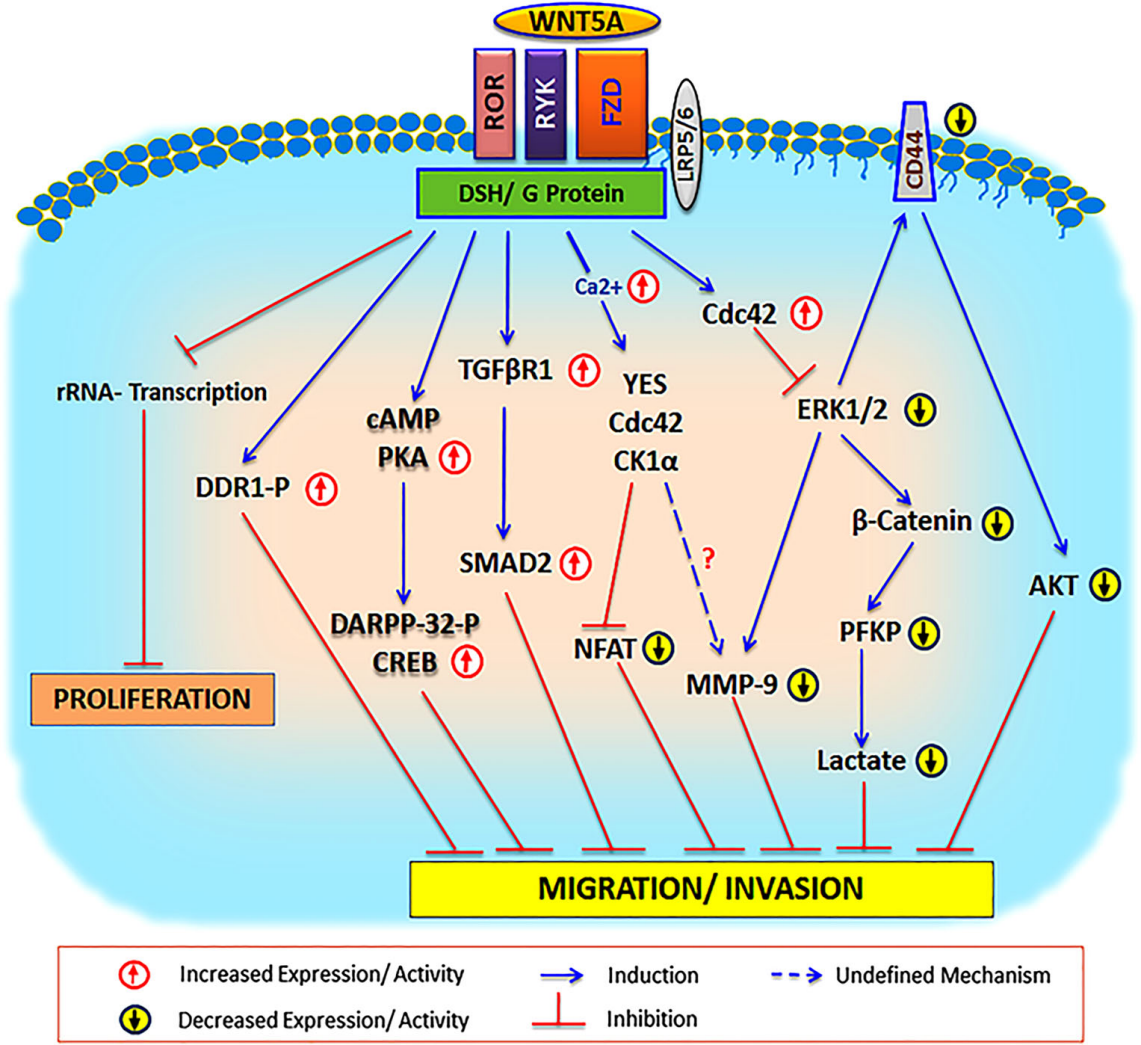
WNT5A signaling and how it mediates tumor-suppressive effects in breast cancer cells. Schematic outlining of how WNT5A, *via* binding to different cognate receptors (e.g., RYK, FZD), mediates downstream signaling cascades and how these affect proliferation, migration and invasion in breast cancer cells. The red up-arrow symbol refers to upregulation or activation of the indicated proteins, whereas the black down-arrow symbol refers to downregulation or inactivation of the indicated signaling molecules. The blue horizontal arrow refers to activation of a signaling molecule, whereas the blue broken arrow indicates undefined signaling mechanisms. Finally, the red stop symbol indicates inhibition of proliferation or migration/invasion

It has been established that WNT5A expression in breast cancer tissue predicts a prolonged disease-free survival, whereas absence or low expression of WNT5A in breast cancer tissue is associated with a shorter disease-free survival [19, 22–25]. However, when it comes to WNT5A expression in tumor-associated macrophages residing in breast cancer tissue and how it relates to prognosis, very little, if any, information is available. Furthermore, there is an unclear picture with regard to the functional relevance of macrophage-derived WNT5A in cancer [45]. Pukrop and co-workers have suggested that macrophage-derived WNT5A is the cause of macrophage-induced invasiveness of breast cancer cells [46]. However, for these experiments, the authors have used a non-invasive luminal breast cancer cell line (MCF-7) that normally has an endogenous WNT5A expression. Moreover, in the same study, these authors observed WNT5A-positive macrophages in 7 out of 17 primary tumor samples. In samples with WNT5A-positive macrophages, such cells only constituted 5–15% of the total number of macrophages in the tumor tissue [46]. In light of the above findings, we postulate that the contribution of macrophage-derived WNT5A might be negligible if WNT5A is expressed in the breast cancer cells. Although from a different cancer type, the finding that macrophage-derived WNT5A causes regression of basal cell carcinoma [47] either indicates a complex action of macrophage-derived WNT5A and/or that our knowledge is limited regarding its functional effect(s). In another study by Yiu and co-workers, it was demonstrated that NFAT promotes invasive migration through Glypican 6 (GPC6). GPC6 was suggested to regulate invasive migration by inhibiting WNT/β-catenin signaling and upregulating the WNT5A/JNK/p38 pathway [30]. However, a major drawback of the study was the use of a WNT5A/WNT5B antibody for the detection of the WNT5A protein. This WNT5A/WNT5B antibody detects endogenous levels of both WNT5A and WNT5B proteins. In another study, Zhu et al. demonstrated that short-term stimulation with rWNT5A increases breast cancer cell migration *via* the Dvl2/Daam1/RhoA signaling axis [48]. WNT5A exposure can trigger an early and transient increase in ERK1/2 activity that might lead to an initial but time-limited increase in cancer cell migration, as shown by Prasad et al. [38]. We believe that in the context of breast cancer progression, prolonged exposure of WNT5A is a more relevant physiological approach. A schematic diagram (Fig. 1) shows the most important WNT5A-triggered signaling pathways believed to participate in the regulation of breast cancer cell migration and invasion.

## WNT5A signaling and breast cancer metabolism: new insight

The oncogenic signaling that occurs in tumor cells often result in altered cellular metabolism, known as the Warburg effect, resulting in aerobic glycolysis irrespective of the presence of oxygen [49]. Furthermore, the WNT/β-catenin pathway has been shown to be involved in the pathogenesis of type 2 diabetes and obesity, which highlights its importance in metabolism and energy expenditure [50]. Although WNT signaling regulates liver metabolism and can stimulate aerobic glycolysis by mTORC2-AKT signaling during osteoblast differentiation, the direct role of WNT5A in the metabolic reprogramming of cancer is still under-investigated [51]. Recently, β-catenin-dependent and β-catenin-independent WNT signaling have been associated with metabolic reprogramming of cancer cells [50]. In breast cancer cells, WNT/Snail signaling induces a glycolytic switch and represses oxidative phosphorylation and cytochrome c oxidase activity [52]. Using two metastatic breast cancer cell lines, i.e., MDA-MB-231 and MDA-MB-468 cells, Prasad et al. demonstrated that WNT5A signaling inhibits lactate secretion, thereby impairing breast cancer cell migration and invasion [53]. Detailed investigation of WNT5A signaling revealed downregulation of the β-catenin-phosphofructokinase-platelet type (PFKP) axis, thereby directly affecting lactate production and migration of breast cancer cells. The authors further established that WNT5A signaling also inhibits monocarboxylate transporter 1 (MCT1) expression, thereby inhibiting lactate uptake in breast cancer cells [53]. These findings were contrary to previous findings in melanoma in which WNT5A acts as a tumor promoter [54]. WNT5A-treated melanoma cells demonstrated an increase in glycolytic activity with concomitant upregulation in lactate dehydrogenase (LDH) activity, the AKT/mTOR pathway and migration. In the same study, the authors demonstrated that WNT5A signaling in breast cancer cells increases oxidative phosphorylation [54].

In breast cancer, reconstitution of WNT5A signaling inhibits crucial components of aerobic glycolysis that result in decreased lactate production and uptake suggest that a loss of WNT5A expression might be involved in a metabolic shift to glycolysis during early breast cancer development. The demonstration that reconstitution of WNT5A signaling impairs breast cancer cell migration and invasion is dependent on an impaired aerobic glycolysis and a restored oxidative phosphorylation strengthens its therapeutic importance in breast cancer cells lacking an endogenous expression of the WNT5A protein.

## Therapeutic targeting of WNT5A signaling

Presence of the WNT5A ligand in human breast cancer correlates with increased disease-free survival and overall survival, indicating that WNT5A functions as a tumor suppressor [19, 22, 23]. The concept that WNT5A acts as a suppressor of breast cancer progression is also supported by the results of numerous *in vitro* studies, indicating that WNT5A primarily reduces the migratory and invasive capacities of breast cancer cells [19, 22–25]. The idea would then be to reconstitute WNT5A signaling in patients who have been diagnosed with breast cancer with low or absent endogenous expression of WNT5A protein in their cancer tissue. A reconstitution of WNT5A signaling in such patients would then serve as a complementary therapy to the presently employed treatment by the ability of the reconstituted WNT5A signaling to specifically impair the dissemination process of such tumors.

The WNT5A molecule is a relatively large and complex protein with numerous cysteine residues. Its activity is dependent on different post-translational modifications [4, 5]. Furthermore, the WNT5A molecule is hydrophobic and has a heparin sulfate-binding domain important for its auto- and paracrine functions that make it less likely that the WNT5A molecule can be used for systemic treatment since it will not reach and target breast cancer cells. Taken together, these properties make the WNT5A molecule unsuitable as a drug candidate. All of these problems could be overcome if one could define a small molecule that mimics the functional responses triggered by the WNT5A molecule.

In search of a WNT5A-mimicking molecule, we collaborated with Dr. Villoutreix (INSERM, Paris). Based on sequence analysis of WNT5A, we identified several peptide sequences predicted to be solvent exposed and thus able to interact with receptor structures exposed on cancer cells. We identified two peptide sequences that had the ability, similar to rWNT5A, to impair migration of breast cancer cells lacking endogenous expression of the WNT5A protein [55]. We continued our study by further investigating the shorter of these two peptides, which contained 12 amino acids. This 12-amino acid peptide was shortened with 2 amino acids at a time from the N-terminal side to investigate how short this peptide could be and still keep its anti-migratory ability in breast cancer cells. When only six amino acids were left, this peptide had lost its functional abilities. However, the N-terminal amino acid in this hexapeptide was a methionine. Based on how bacteria synthesize their proteins, we decided to formylate this N-terminal methionine. This modification regained the functional WNT5A-mimicking activity of this hexapeptide [55]. The fact that small formylated peptides released from bacteria at the site of an infection are functionally active in an environment of low pH and high protease activity suggested to us that our formylated WNT5A-derived hexapeptide (Foxy5) would similarly resist degradation in the tumor microenvironment, a site also likely to have a low pH and elevated protease activity.

**Fig. 2:**
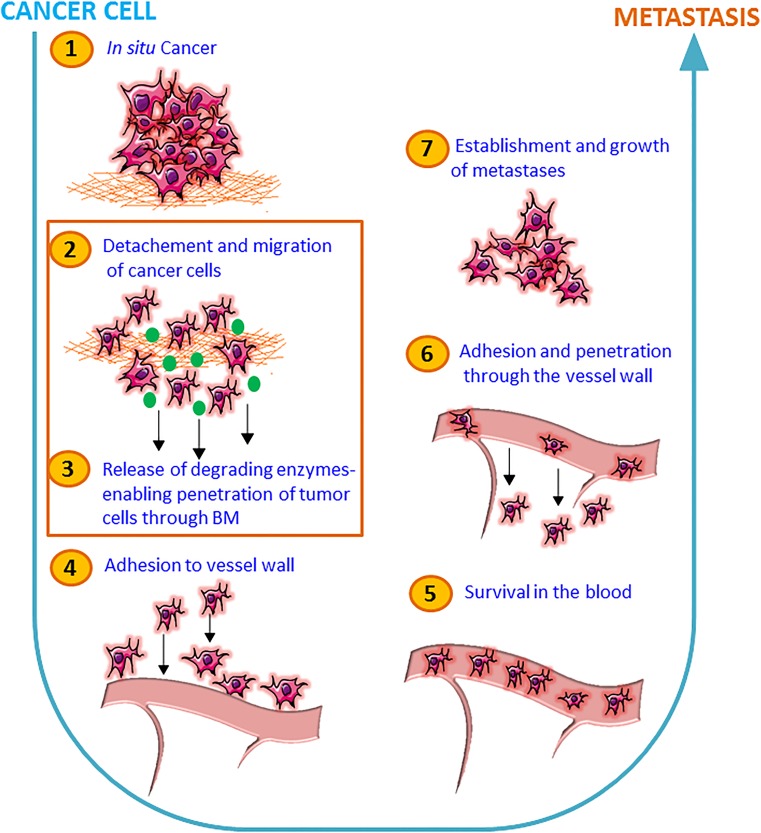
Anti-metastatic effects of Foxy5 during the initial stages of metastasis. A schematic drawing that presents a simplified and step-wise (steps 1 to 7) cartoon of the metastatic processes. We have highlighted the inhibitory effect of Foxy5 on two important and initial events of metastatic process, i.e., detachment and subsequent migration of cancer cells (step no. 2) and next release of degrading enzymes thereby enabling the tumor cells to penetrate through the basement membrane (BM) (step no. 3)

**Fig. 3:**
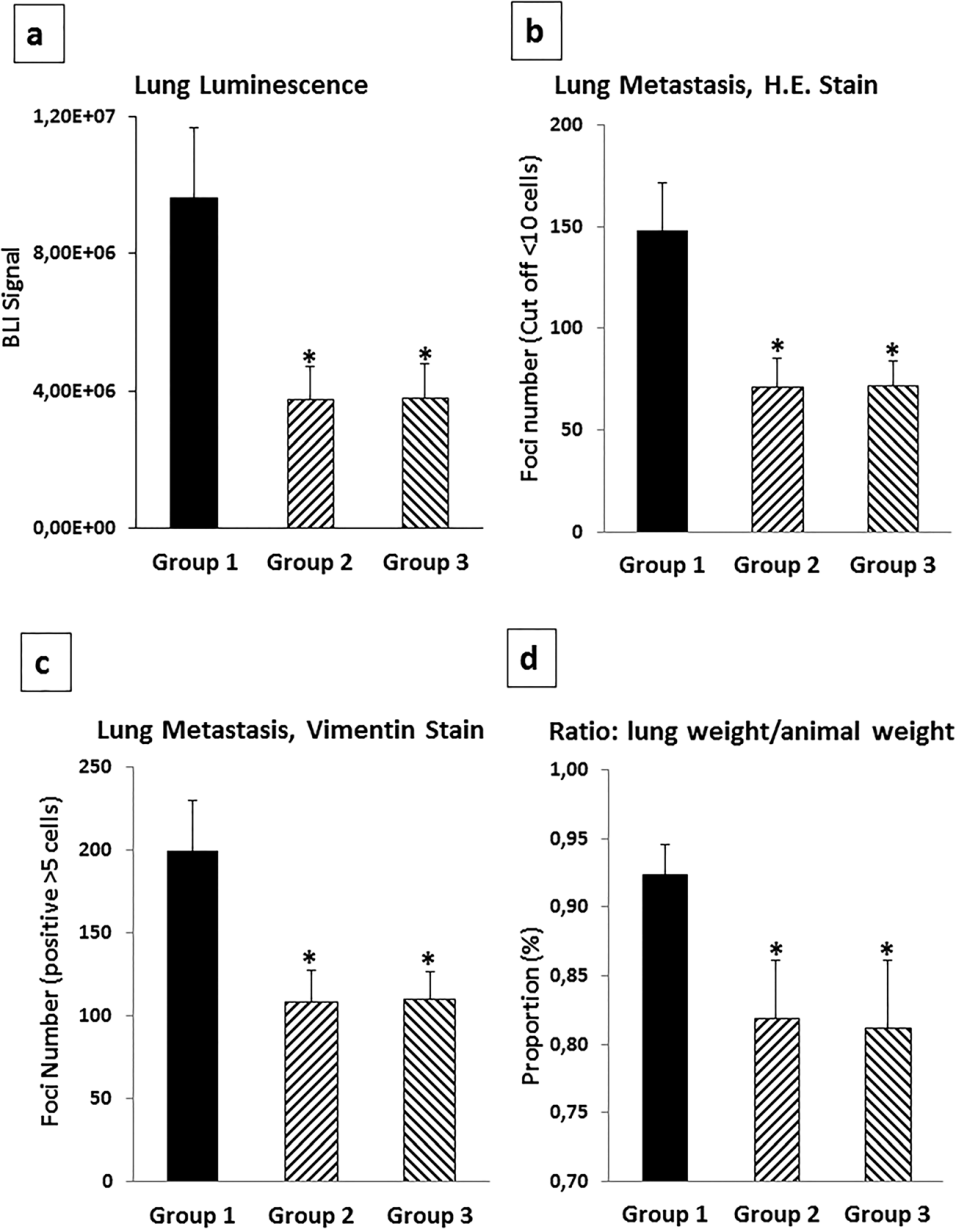
Foxy5 inhibits lung metastasis of breast cancer cells directly injected into the blood stream. Animal experiments were conducted to evaluate a possible anti-metastatic effect of Foxy5 on breast cancer cells injected into the blood stream *via* the tail vein of nude mice. The animals were injected with luciferase-labeled MDA-MB-231-LM2-4175 breast cancer cells and divided into three different groups depending on how they were treated with either vehicle or Foxy5. The vehicle-treated animals refer to group 1, the animals treated with Foxy5 represent group 2, and group 3 represent the animal where the MDA-MB-231-LM2-4175 breast cancer cells were first pre-treated with Foxy5 *in vitro* before inoculated into the tail vein and then treated with Foxy5 as the mice in group 2. The amounts of breast cancer lung metastases were determined by four different means to properly evaluate the anti-metastatic effect of Foxy5. The lung metastasis was evaluated through **a** lung BLI, **b** H.E. staining, **c** Vimentin staining, and **d** ratio: lung weight/animal weight

**Fig. 4:**
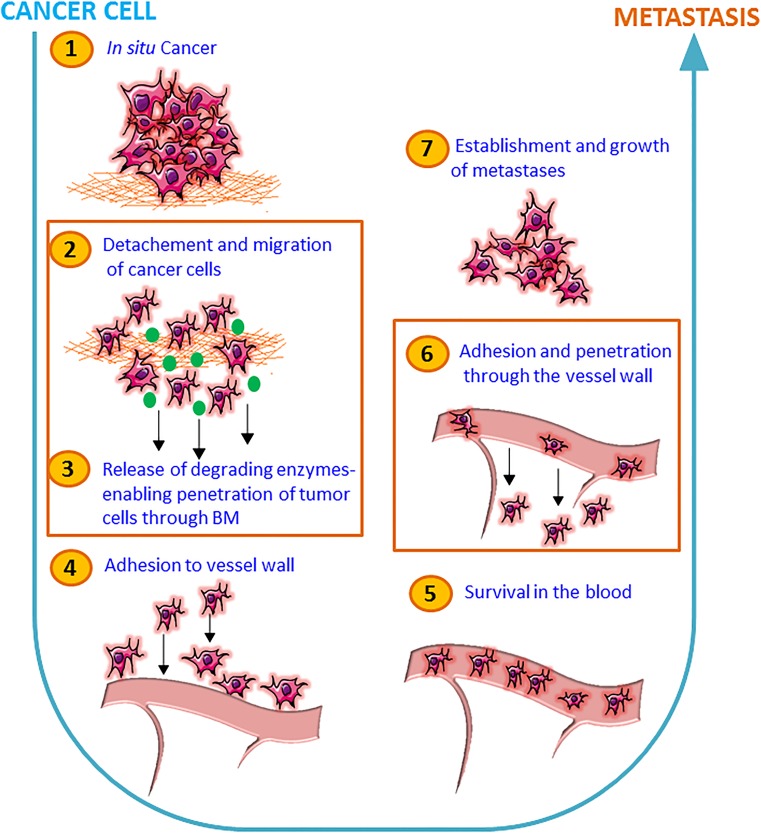
Foxy5 targets initial as well as later metastatic stages during breast cancer progression. The schematic diagram is outlining various steps (1–7) involved in cancer cell metastasis. In this figure, we have updated and high-lighted that Foxy5 could indeed target three important steps of cancer cell metastasis, i.e., detachment and subsequent migration of cancer cells (step no. 2), next release of degrading enzymes thereby enabling the tumor cells to penetrate through the basement membrane (BM) (step no. 3), and also adhesion to and migration through the vessel wall (extravasation) (step no. 6)

In subsequent animal experiments with WNT5A-negative 4T1 breast cancer cells inoculated orthotopically in mammary fat pads, we were able to show that treatment every 4th day with Foxy5 for up to 24 days significantly reduced the metastatic spread by 70–80% [56]. In these experiments, we started the Foxy5 treatment the same day as the tumor cells were injected into the mammary fat pads. This prompt treatment with Foxy5 and the trend of Foxy5 to increase the primary tumor volume in the mice [56] were the reason why our initial working hypothesis, outlined in Fig. 2, was that Foxy5 affected early events in the metastatic process (steps no. 2 and 3). Consequently, at this stage, our working hypothesis was that Foxy5 acts at the first steps of the metastatic process and that it is hindering the tumor cells to spread from the primary tumor site by counteracting step number 2 and 3 in the schematic drawing as outlined in Fig. 2. However, it cannot be excluded that Foxy5 could also affect later steps in the metastatic process. Foxy5 and its anti-metastatic potential were patented and a small Swedish biotech company named WntResearch AB that has since worked on Foxy5’s clinical development now holds the patents. The company first performed pre-clinical toxicological studies in preparation for clinical phase 1 studies; these revealed no major adverse effects. The *in vivo* study revealing an anti-metastatic effect of Foxy5 in breast cancer models was performed after inoculation of the tumor cells into the mammary fat pads and prior to the metastatic spread of the disease [56]. Therefore, another question is if Foxy5 will differently affect the metastatic process in patients with already established metastatic disease and with a high likelihood to have circulating tumor cells. To experimentally test this issue, we injected human MDA-MB-231-LM2-4175 TNBC cells directly into the tail vein of immune-deficient mice and started the treatment of these animals with Foxy5 directly after this and then every 2nd day for 13 days. These human breast cancer cells originated from Dr. Massagué’s laboratory and have been enriched for their ability to metastasize to the lungs and have also been luciferase-labeled to enable detection of metastatic foci [57]. Pharmatest Services Ltd. (Turku, Finland) was contracted to conduct these animal experiments and after the termination of the experiments sent primary cancer tissue together with the lungs of the animals for immunohistochemical analyses in our lab. To avoid the difficulty of proper solution of Foxy5, the company received Foxy5 lyophilized in TRIS salts. The results (Fig. 3) show that in comparison with vehicle-treated animals (group 1), there was an approximately 50% reduction in lung metastasis if the animals were treated with Foxy5 (group 2). The reason for choosing vehicle-treated animals as controls is our observation that a formylated control peptide had a high risk of triggering activation of macrophages that would cause an increased metastatic spread. Such an effect would then result in an erroneous too large anti-metastatic effect of Foxy5. This effect was not increased if the cells were also pre-treated with Foxy5 *in vitro* before being inoculated into the tail veins and the animals treated with Foxy5 (group 3). These data indicate that Foxy5 impairs breast cancer metastasis at two different sites of the metastatic process. This is now outlined in the supplemented schematic outline, where Foxy5 is shown to interfere with the metastatic process of breast cancer (Fig. 4). These findings are in good agreement with those published by Jiang and co-workers, who worked with WNT5A-expressing MDA-MB-231 and 4T1 breast cancer cells exhibiting reduced cell migration (but normal cell growth) compared to their respective control cells not expressing WNT5A [9]. Most importantly, in the present context, the authors demonstrated significant reductions in lung metastasis when WNT5A-expressing 4T1 cells were injected *via* the tail vein compared to 4T1-control cells lacking WNT5A expression.

Patients included in a phase 1 clinical studies of oncological drug candidates are normally those with advanced metastatic cancer. This means that an anti-metastatic effect of Foxy5 cannot be evaluated in the patients included in any of the company’s phase 1 studies. The first phase 1 clinical study of Foxy5 has been completed (www.clinicaltrials.gov; NCT02020291) and it revealed no major adverse reactions to this drug candidate. This is very good from the perspective of combining Foxy5 treatment with the present cytotoxic drug treatments but it imposes a problem in that no maximal tolerable dose could be defined and consequently the data could not be used for defining the dose for a subsequent phase 2 study. In order to define such a dose for a phase 2 study, a phase 1b study with a different design had to be performed (www.clinicaltrials.gov; NCT 02655952). In this phase 1b study, blood samples and tumor biopsies will be taken before and after the treatment with Foxy5 and a suitable dose for a phase 2 study will be chosen based on analyses of its biological activity. Once a dose has been decided, one could then evaluate the efficacy of Foxy5 in combination, sequentially or simultaneously, with conventional therapies in the treatment of cancer patients with a low or no expression of the WNT5A protein in their primary tumor. This would be particularly useful for breast cancer patients that harbor a high risk of progressing to metastatic disease, such as TNBC patients. To effectively stop tumor metastasis, a future anti-metastatic complementary treatment should be introduced as soon as possible after diagnosed with breast cancer.

Once the phase 1b study has been completed, the dose for the phase 2 study will be decided. The phase 2 study will be performed in patients who lack expression of WNT5A protein or exhibit a low level of WNT5A protein expression in their primary tumors and who have no distal metastases at the time at which the Foxy5 treatment is initiated. Such a phase 2 study will finally answer the question of whether Foxy5 has the same anti-metastatic effect in humans as it has been shown to have in animal experiments [56, 58].

### Safety issues with Foxy5

We have not noted any adverse effects of treating mice with Foxy5 in any of the *in vivo* experiments, in the present study or our previous studies [56, 58]. These findings have been confirmed in pre-clinical toxicological studies in rats and dogs and in the clinical phase 1 studies performed on cancer patients. This lack of toxicity of Foxy5 will make it easier to combine, sequentially or simultaneously, with presently used chemotherapies or targeted cancer therapies that can have substantial toxic side effects. Another safety issue with Foxy5 is based on the findings that WNT5A promotes tumor progression in tumors such as melanoma, oral squamous cell carcinoma, and gastric cancer [18]. Although a limited number of patients diagnosed with breast cancer could have a history of tumors such as melanoma, squamous cell carcinoma, or gastric cancer, such patients should not be treated with Foxy5. In the performed phase 1 studies, patients with a previous history of such tumors were excluded from participating in the study.

### Conclusions

Numerous studies of how WNT5A signaling affects cancer cell behavior, *in vitro*, have successfully been performed with rWNT5A. It has been shown that treatment of invasive breast cancer cells with rWNT5A increase their adhesion resulting in a reduction of their migration and invasion. However, the development of WNT5A as a therapeutic target for the treatment of breast cancer patients has been impeded by the fact that WNT5A has a specific heparin sulfate-binding domain that will hinder its distribution *in vivo*. Furthermore, the WNT5A molecule undergoes several different post-translational modifications required for its biological activity making it less attractive as a drug candidate. Therefore, it was of interest to find a molecule mimicking the tumor suppressor effects of WNT5A on breast cancer cells that could also be used *in vivo*. We therefore screened for a WNT5A-mimicking peptide and finally, after screening several peptide sequences, we identified a formylated six amino acid peptide named Foxy5 that was found to mimic the signaling and functional effects of rWNT5A on breast cancer cells. In good agreement with our assumption, it was demonstrated that treatment of WNT5A-lacking breast cancer cells with Foxy5 reduces the metastatic spread of breast cancer cells by 70–80% in an orthotopic mouse model, highlighting its therapeutic potential as an anti-metastatic drug candidate. Interestingly, in a recent study from our lab, WNT5A and its mimic Foxy5 has been shown to reduce breast cancer cell migration even in the presence of high lactate levels, suggesting its therapeutic potential in managing highly glycolytic breast tumors as well. The majority of *in vitro* and *in vivo* experiments on breast cancer cells have been performed on TNBC cell lines and clinical studies on TNBC patients revealed that they have an increased percentage (around 75%) of tumors with no or low expression of WNT5A protein, which might be the reason why they are highly metastatic and associated with early relapse. These findings provide a challenge in taking the drug candidate Foxy5 into the clinic and test its anti-metastatic efficacy in breast cancer patients and then in particular TNBC patients.

## Abbreviations

DARPP-32: Dopamine and cAMP-regulated phosphoprotein of 32 KDa
DDR1: Discoidin domain receptor-1
DVL1: Segment polarity protein disheveled homolog
ELAV: Embryonic lethal abnormal vision
EMT: Epithelial mesenchymal transition
Foxy5: Formylated WNT5A-derived hexapeptide
FZD: Frizzled receptors
GPI: Glycosylphosphatidylinositol
GPC6: Glypican 6
HGF: Hepatocyte growth factor
HSPGs: Heparin sulfate proteoglycans
IDCs: Invasive ductal carcinoma
LDH: Lactate dehydrogenase
LRP: Low-density lipoprotein receptor-related protein co-receptors
MCT1: Monocarboxylate transporter 1
NFAT: Nuclear factor associated with T cells
PFKP: Phosphofructokinase-platelet type
RB: Retinoblastoma protein
ROR: Receptor tyrosine kinase-like orphan receptor family
rWNT5A: Recombinant WNT5A
RYK: Related to receptor tyrosine kinase
TGF-β: Transforming growth factor-β
TGFβR1: Transforming growth factor β-receptor 1
TIC: Tumor-initiating cells
TNBC: Triple-negative breast cancer
WNT: Wingless-type MMTV integration site
WNT5A-L: Long WNT5A mRNA isoform
WNT5A-S: Short WNT5A mRNA isoform
